# Hemodynamics of a right ventricular outflow tract aneurysm assessed with 4D flow MRI before and after unilateral pulmonary artery balloon angioplasty: A case report

**DOI:** 10.1016/j.radcr.2025.04.083

**Published:** 2025-05-15

**Authors:** Hideharu Oka, Kouichi Nakau, Keita Ito, Rina Imanishi, Kazunori Fukao, Sadahiro Nakagawa, Tatsuya Suzuki, Satoru Takahashi

**Affiliations:** a Department of Pediatrics, Asahikawa Medical University, Hokkaido, Japan; b Section of Radiological Technology, Department of Medical Technology, Asahikawa Medical University Hospital, Hokkaido, Japan

**Keywords:** Congenital heart disease, Case report, 4D flow MRI, Right ventricular outflow tract aneurysm, Vortex flow

## Abstract

Right ventricular outflow tract aneurysms are rare postoperative complications associated with congenital heart diseases. The hemodynamics associated with a right ventricular outflow tract aneurysm serve as important risk indicators for aneurysm expansion and thrombus formation; however, they have not been thoroughly evaluated. A 3-year-old girl was diagnosed with Double-outlet right ventricle who underwent surgical correction with patch enlargement of the right ventricular outflow tract at 2.5 years. Postoperatively, a patch expansion mass was observed and 4D flow MRI showed vortex flow. Notably, vortex flow within the aneurysm persisted even after balloon dilation of the unilateral pulmonary artery. Evaluating blood flow in right ventricular outflow tract aneurysms is crucial for determining prognosis and guiding treatment strategies.

## Introduction

Tetralogy of Fallot (TOF) and double-outlet right ventricle (DORV) are congenital heart diseases that frequently require surgical intervention of the right ventricular outflow tract (RVOT). An RVOT aneurysm has been reported as a rare postoperative complication [[1], [2], [3], [4], [5], [6], [7], [8], [9]]. The contributing factors include pressure overload due to residual RVOT stenosis, the size of the patch used for repair, and any remaining ventricular septal defects [1]. The hemodynamics associated with an RVOT aneurysm serve as important risk indicators for aneurysm expansion and thrombus formation; however, they have not been thoroughly evaluated.

In this case report, we describe the use of 3D cine phase-contrast magnetic resonance imaging (4D flow MRI) to assess the hemodynamics within an RVOT aneurysm that developed in a 3-year-old girl after DORV repair and to evaluate the changes in flow dynamics following balloon dilation of the left pulmonary artery (PA) stenosis. Our findings aim to clarify how hemodynamic changes might influence future treatment strategies for RVOT aneurysms.

## Case presentation

**Fig. 1:**
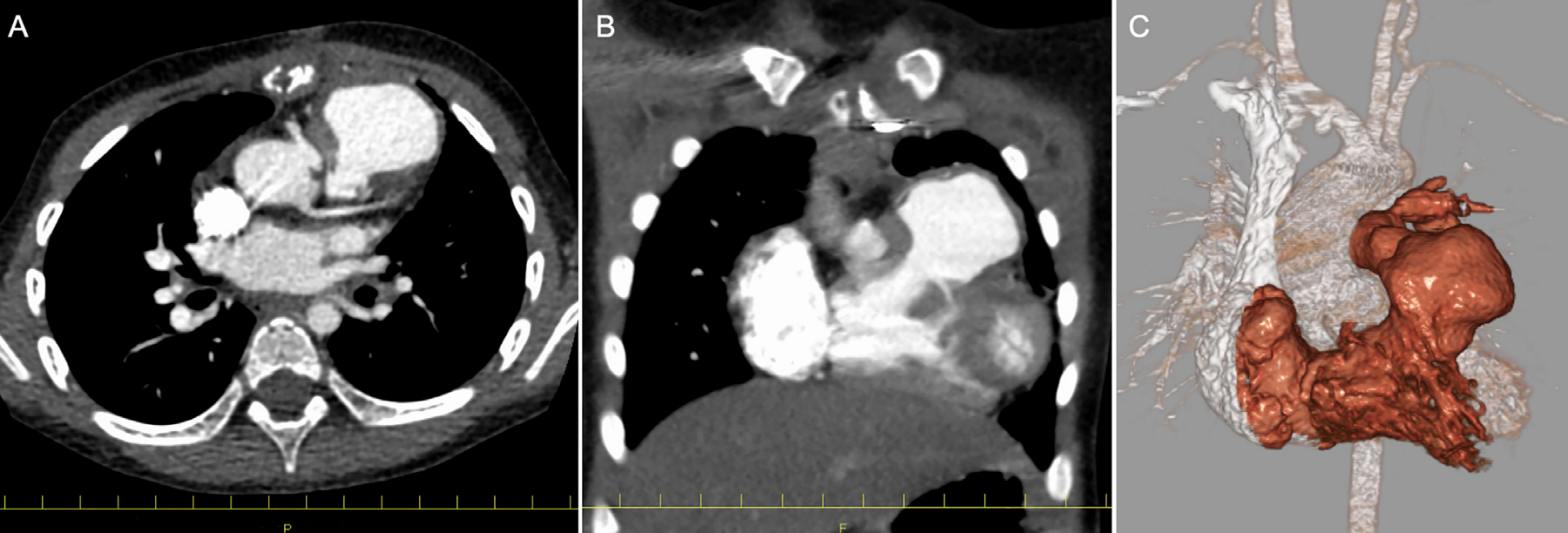
(A-C) Contrast-enhanced computed tomography revealed aneurysm formation in the RVOT.

A 3-year-old girl was diagnosed with DORV in utero and was born at 38 weeks and 3 days of gestation. After birth, she was found to have a bicuspid PA valve and subvalvular pulmonary stenosis, which prompted regular follow-up. At 1 month of age, her oxygen saturation decreased to 85%, and echocardiography revealed a PA blood flow velocity of 4 m/s. Consequently, beta-blocker therapy was initiated. Despite this intervention, her oxygen saturation continued to decline, leading to the decision to perform a right-sided modified Blalock–Taussig shunt surgery at 2 months of age. After the patient gained weight, surgical correction was performed at 2 years and 6 months of age. The ventricular septum was closed using an expanded Polytetrafluoroethylene patch and patch enlargement of the RVOT following resection of the myocardium on the free wall side. The PA valve was repaired through commissurotomy, and both PA were expanded with artificial vessels. During the postoperative follow-up, an RVOT aneurysm and left PA stenosis were identified. Consequently, the patient was admitted for further examination at the age of 3 years. Contrast-enhanced computed tomography revealed an aneurysm in the RVOT (Fig. 1A–C). No thrombus was found in the aneurysm. Cardiac catheterization indicated a right ventricular (RV) systolic and end-diastolic pressure were 57 and 7 mmHg, a pressure gradient of 14 mmHg between the left PA and the main PA, and a pressure gradient of 27 mmHg between the main PA and the RV. A left PA stenosis was measured at 4.7 mm. The aneurysm in the RVOT measured 3.3 × 2.2 cm. No residual ventricular septal defects were observed. Cardiac MRI results as RV function were RVEDVI 67.3ml/m^2^, RVESVI 44.2ml/m^2^, RVEF 35%, RVOT 2.6m/s, and pulmonary regurgitation 3%.

**Fig. 2:**
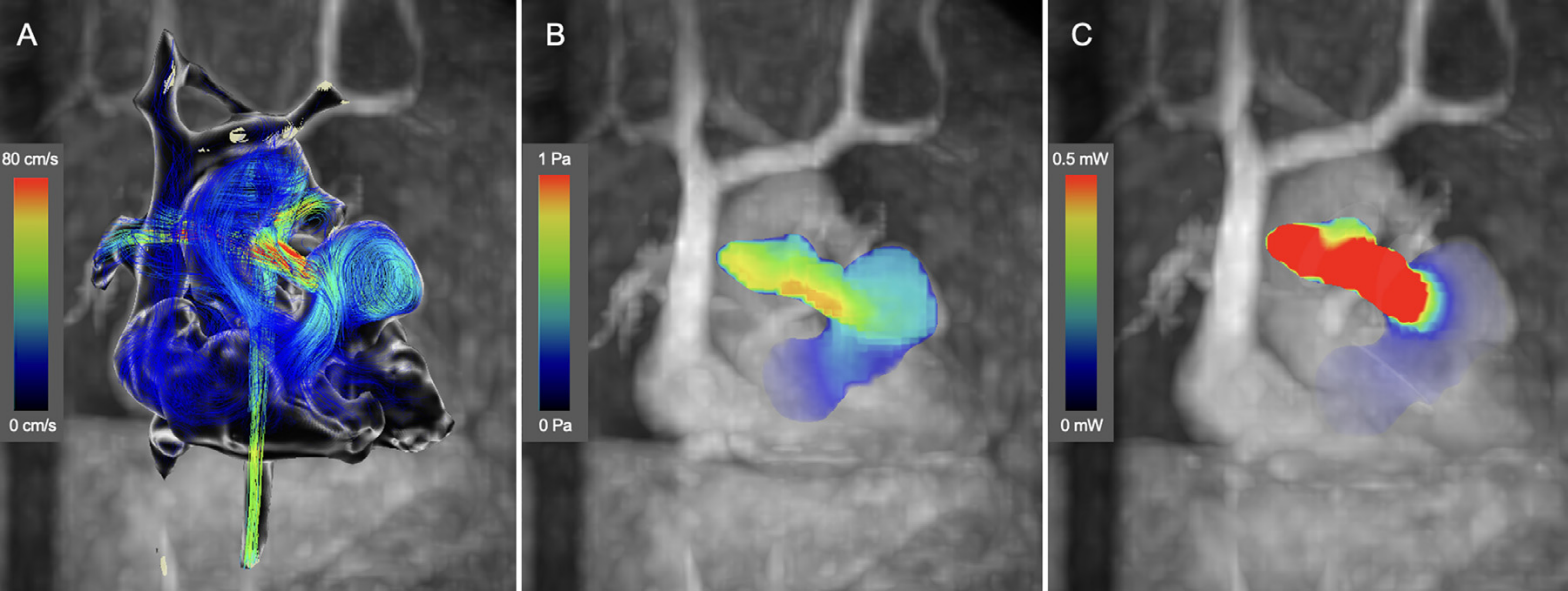
(A-C) Preinterventional 4D Flow MRI. (A) The streamline shows marked vortex flow at the aneurysm of the RVOT in the systolic phase. (B) A peak WSS of 0.18 Pa and an average WSS of 0.09 Pa. (C) A peak EL of 0.01 mW and an average EL of 0.01 mW within the RVOT aneurysm.

After obtaining written informed consent, the Institutional Review Board approved the 4D-flow MRI study (IRB No. 19250). The scan parameters were summarized in Table 1. The analysis was performed using a workstation (Cvi42; Circle Cardiovascular Imaging, Calgary, Canada). The 4D flow MRI revealed a significant vortex flow within the aneurysm during the systolic phase, with a peak wall shear stress (WSS) of 0.18 Pa and an average WSS of 0.09 Pa, and a peak energy loss (EL) of 0.01 mW and an average EL of 0.01 mW within the RVOT aneurysm (Fig. 2A–C; Supplementary Video 1). The peak EL for the RVOTS portion was 2.67 mW and the average EL was 0.58 mW. Pulmonary blood flow scintigraphy indicated a blood flow ratio of 72:28 between the right and left PA.

**Table 1:** 4D flow MRI scan parameter.

Scanner	Siemens MAGNETOM Vida 3 Tesla
Acceleration method	GRAPPA 3
Field of view (mm)	340 × 340
Acquired voxel size (mm^3^)	2.0 × 2.0 × 2.0
Echo spacing /Echo time (msec)	4.96/2.79
Temporal resolution (msec)	41
Flip angle (°)	8
Cardiac phases	25-30
Segment	2
VENC (cm/s)	150
Cardiac gating	retrospective ECG gating
Free-breathing cardiorespiratory synchronized	(+)
Scan time (min)	15

**Fig. 3:**
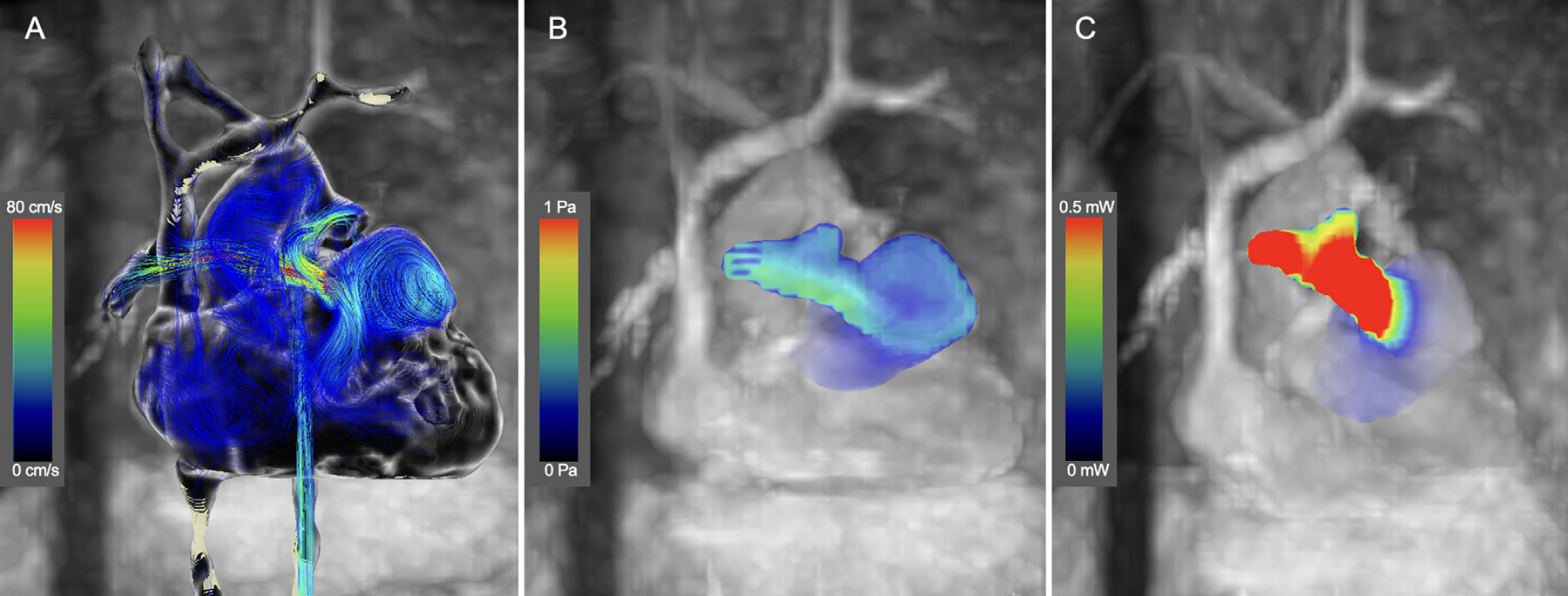
(A-C) Postinterventional 4D Flow MRI. (A) The streamline shows residual vortex flow at the aneurysm. (B) The peak WSS was 0.16 Pa, the average WSS was 0.09 Pa. (C) A peak EL of 0.33 mW and an average EL of 0.17 mW. The WSS and EL within the RVOT aneurysm was unchanged compared to before balloon dilation.

Balloon dilation of the left PA stenosis was performed to prioritize noninvasive treatment. A balloon with a diameter of 10 mm and length of 20 mm was used, and the left PA was dilated until the waist disappeared. Following the procedure, the RV systolic and end-diastolic pressure were 50 and 4 mmHg, and the pressure gradient between the left and main PA improved to 4 mmHg. A left PA stenosis was measured at 7.0 mm. Cardiac MRI results as RV function were RVEDVI 79.6 mL/m^2^, RVESVI 40.7 mL/m^2^, RVEF 48%, RVOT 2.7m/s, and pulmonary regurgitation 3%. A 4D flow MRI conducted the day after treatment showed a slight reduction in vortex flow within the aneurysm. The peak WSS was 0.16 Pa, while the average WSS remained at 0.09 Pa, and a peak EL of 0.33 mW and an average EL of 0.17 mW within the RVOT aneurysm indicating that the overall WSS and EL was unchanged (Fig. 3A–C; Supplementary Video 2). The peak EL for the RVOTS portion was 2.07 mW and the average EL was 0.52 mW. Pulmonary blood flow scintigraphy indicated that the blood flow ratio between the right and left PA improved to 52:48. The girl is now 3.5 years old and in good health, with no enlargement of the RVOT aneurysm observed. We continued to monitor her closely while considering the appropriate timing for intervention for the RVOT aneurysm.

## Discussion

We conducted 4D flow MRI before and after balloon dilation in a patient with an RVOT aneurysm following DORV repair to assess the hemodynamics within the aneurysm. The 4D flow MRI revealed significant vortex flow within the RVOT aneurysm. This vortex flow persisted after balloon dilation, suggesting that the patient was at risk for future aneurysm enlargement.

RVOT aneurysms are a rare complication that can occur in cases requiring intervention in the RVOT [[1], [2], [3], [4], [5], [6], [7], [8], [9]]. Because most of these patients undergo reoperation shortly after diagnosis, there are few reports on the hemodynamics of RVOT aneurysm before and after treatment. The 4D flow MRI evaluation before balloon dilation revealed significant vortex flow within the aneurysm, which is considered a risk factor for aneurysmal enlargement in the aorta [10]. On the other hand, vortex flow is believed to prevent stagnation of blood flow within the aneurysm. Therefore, vortex flow helps prevent thrombus formation in the left atrium, while the loss of this vortex flow is associated with an increased risk of thrombus formation [11]. Therefore, it is crucial to monitor for thrombus formation if the vortex flow is reduced. In the present case, the vortex flow within the aneurysm slightly diminished after balloon dilation. While this reduction may contribute to a decreased risk of aneurysm enlargement, the persistence of vortex flow indicates that the risk of future aneurysm enlargement still exists. The lack of change in WSS and EL due to RV pressure decompression may also indicate residual load on the aneurysm. Elevated EL has been reported to be associated with major adverse cardiovascular events [12]. In this case, only LPS was treated in consideration of patient invasiveness, but the finding of high EL in RVOT may suggest that treatment of RVOT stenosis may be considered at an early stage in the future. Additionally, as mentioned earlier, a decrease in vortex flow is believed to increase the risk of thrombus formation. Consequently, it remains unclear whether reducing vortex flow is advantageous for patients. Further studies are needed to establish the level of vortex flow that prevents both aneurysm enlargement and thrombus formation. However, when vortex flow is pronounced, as in this case, the risk of aneurysm enlargement is considered sufficiently high to warrant RVOT reconstruction.

## Conclusion

Our 4D flow MRI study of an RVOT aneurysm after DORV repair revealed the persistence of vortex flow even after balloon dilation. While the reduction in vortex flow after treatment may suggest a decreased risk, the continued presence of vortex flow requires close monitoring. Further research is needed to better understand the optimal management of RVOT aneurysms and the role of vortex flow in predicting long-term outcomes.

## Patient consent

Written informed consent was obtained from the parents for publication of this case report in line with COPE guidance.

## Ethics approval and consent to participate

This study was conducted in compliance with the standards of the Declaration of Helsinki and the current ethical guidelines and was approved by our institutional ethics board (Approval Number 19250). Written informed consent was obtained from the participant.

## Appendix Supplementary materials

Preinterventional 4D Flow MRI. Image, video 1

Postinterventional 4D Flow MRI. Image, video 2

## References

[1] Seybold-Epting W., Chiariello L., Hallman G.L., Cooley DA. (1977). Aneurysm of pericardial right ventricular outflow tract patches. Ann Thorac Surg.

[2] Rosenthal A., Gross R.E., Pasternac A. (1972). Aneurysms of right ventricular outflow patches. J Thorac Cardiovasc Surg.

[3] Tomasulo C.E., Ravishankar C., Natarajan S., Mascio C.E., Glatz AC. (2021). Large aneurysms and pseudoaneurysms of surgically reconstructed right ventricular outflow tracts. Cardiol Young.

[4] Rato J., Ataíde R., Teixeira A. (2020). Giant pseudo-aneurysm of the right ventricular outflow tract after tetralogy of Fallot repair. Cardiol Young.

[5] Mack T., Vachon T., Boswell G. (2011). Right ventricular outflow tract pseudoaneurysm: two cases. J Cardiovasc Comput Tomogr.

[6] Ugurlu B., Oto O., Unal N., Akcoral A. (2001). Pseudoaneurysm of the right ventricular outflow tract complicating balloon dilatation for tetralogy of fallot. Pediatr Cardiol.

[7] Calabrò R., Santoro G., Pisacane C., Pacileo G., Russo M.G., Vosa C. (1999). Repeat syncopal attacks due to postsurgical right ventricular pseudoaneurysm. Ann Thorac Surg.

[8] Peer S.M., Bhat P.S., Furtado A.D., Chikkatur R. (2012). Right ventricular outflow tract aneurysm with thrombus. Interact Cardiovasc Thorac Surg.

[9] Pillai S.K., Reddy H.P., Kulkarni S., Murthy K.S., Cherian KM. (2004). Pseudoaneurysm of homograft placed in right ventricular outflow tract. Ann Thorac Surg.

[10] Terada M., Takehara Y., Isoda H., Wakayama T., Nozaki (2022). Technical background for 4D flow MR imaging. Magn Reson Med Sci.

[11] Sekine T., Nakaza M., Matsumoto M., Ando T., Inoue T., Sakamoto S.I. (2022). 4D Flow MR imaging of the left atrium: what is non-physiological blood Flow in the cardiac system?. Magn Reson Med Sci.

[12] Shiina Y., Nagao M., Itatani K., Shimada E., Inai K. (2024). 4D flow MRI-derived energy loss and RV workload in adults with tetralogy of Fallot. J Cardiol.

